# Targeting Kynureninase Attenuates Radiation-Induced Intestinal Injury via MAPK Signaling Suppression

**DOI:** 10.1155/mi/7023259

**Published:** 2025-09-19

**Authors:** Qingxie Liu, Zhi Ling, Yue Zhu, Weijuan Gong, Guotao Lu, Wei Li, Weixuan Yang, Weiming Xiao, Yaodong Wang

**Affiliations:** ^1^Department of Gastroenterology, Yangzhou Key Laboratory for Precision Treatment of Refractory Bowel Diseases, Affiliated Hospital of Yangzhou University, Yangzhou University, Yangzhou 225001, China; ^2^Department of Oncology, Affiliated Hospital of Yangzhou University, Yangzhou University, Yangzhou 225001, China; ^3^Department of Gastroenterology, Traditional Chinese Medicine Hospital of Kunshan, Suzhou Key Laboratory of Integrated Traditional Chinese and Western Medicine of Digestive Diseases, Kunshan Affiliated Hospital of Yangzhou University, Kunshan, China; ^4^Faculty of Pharmaceutical Sciences, Toho University, Funabashi 274–8510, Chiba, Japan; ^5^Department of Gastroenterology, Huai'an Hospital Affiliated to Yangzhou University (The Fifth People's Hospital of Huai'an), Yangzhou University, Huai'an, Jiangsu, China

**Keywords:** carbidopa, kynureninase, MAPK signaling pathway, radiation-induced intestinal injury

## Abstract

Kynureninase (KYNU), a key enzyme in the tryptophan–kynurenine metabolic pathway, has been increasingly recognized for its role in immune regulation and inflammation. However, its involvement in radiation-induced intestinal injury (RIII) has not been fully elucidated. In this study, we identified a significant upregulation of KYNU expression in the colonic tissues of mice with RIII using transcriptomic analysis and experimental validation. Functional assays demonstrated that KYNU knockdown in NCM460 human intestinal epithelial cells attenuated radiation-induced apoptosis and oxidative stress, while promoting cell proliferation. Mechanistically, RNA sequencing (RNA-seq) and pathway enrichment analyses revealed that KYNU regulates the mitogen-activated protein kinase (MAPK) signaling pathway, as KYNU silencing reduced the phosphorylation levels of key MAPK proteins (extracellular signal-regulated kinase [ERK], c-Jun N-terminal kinase [JNK], and p38) following irradiation. Importantly, pharmacological inhibition of KYNU using carbidopa (CBP) significantly mitigated radiation-induced epithelial injury in vitro. In the RIII mouse model, CBP administration (prevention and treatment) increased the number of crypts, improved intestinal epithelial structure, and maintained the integrity of the intestinal barrier. These findings demonstrate, that KYNU plays a critical role in the pathogenesis of RIII and that its inhibition confers protection against intestinal damage by suppressing MAPK-mediated inflammatory responses. Targeting KYNU may thus offer a promising therapeutic strategy for the prevention and treatment of RIII.

## 1. Introduction

Radiation-induced intestinal injury (RIII) is a common and severe gastrointestinal complication associated with radiotherapy for abdominal, pelvic, and retroperitoneal malignancies. Approximately 90% of patients experience acute gastrointestinal symptoms, such as abdominal pain, diarrhea, and bloating, within 3 months of abdominal and pelvic radiotherapy. Moreover, approximately 50% of patients develop chronic gastrointestinal symptoms, including fibrosis, obliterative vasculitis, and atrophy, within a year after therapy. These complications not only severely impair patients' quality of life but also compromise the effectiveness of oncologic treatment. Although, the pathogenesis of RIII remains obscure and has been associated with epithelial damage, vascular system damage, and intestinal immune and microbial disorders [1, 2]. However, effective and standardized preventive or therapeutic strategies for RIII remain lacking in clinical practice.

Kynureninase (KYNU) is a pyridoxal phosphate-dependent hydrolase that catalyzes a key step in the kynurenine pathway of tryptophan metabolism, thereby facilitating the biosynthesis of nicotinamide adenine dinucleotide (NAD^+^) cofactors [3]. KYNU is ubiquitously expressed in multiple organs, with particularly high expression levels observed in the liver, bladder, and appendix. Beyond its role in cancer metabolism, KYNU has also been implicated in the pathogenesis of various inflammatory and cardiovascular diseases through diverse signaling mechanisms [4]. Notably, recent studies have highlighted the involvement of KYNU in inflammatory bowel diseases, including ulcerative colitis, underscoring its potential role in gut inflammation [5]. However, KYNU's function in RIII remains largely unexplored. In this study, we aimed to investigate the role of KYNU in the development of RIII and elucidate the underlying molecular mechanisms.

## 2. Materials and Methods

### 2.1. Laboratory Animals

The Ethics Committee of the Affiliated Hospital of Yangzhou University approved this study (Approval Number 2023-YKL11-Ke 06), which complied with the principles of the Declaration of Helsinki (2013 version). Thirty-two male C57BL/6 mice (age: 8 weeks and weight: 20–25 g) were procured from the Translational Medical College of Yangzhou University (Yangzhou, Jiangsu, China). The mice were housed under the following conditions: temperature, 20–25°C; humidity, 50%–60%; 12 h light/dark cycle; and ad libitum access to water and standard rodent food. Cage locations were randomized to minimize potential confounders, handling, and measurements. Only the study designer was aware of the group assignments.

### 2.2. Reagents and Antibodies

Reagents were obtained from the following respective suppliers: carbidopa (CBP; Sigma-Aldrich Corp, St. Louis, MO, USA); anti-β-actin, anti-c-Jun N-terminal kinase (JNK), anti-phospho-JNK, anti-phospho-p38, anti-p38, anti-phospho-extracellular signal-regulated kinase (ERK), anti-ERK, anti-Occludin, anti-ZO-1, anti-mouse, and anti-rabbit antibodies (Cell Signaling Technology, Danvers, MA, USA); anti-KYNU antibodies (Wuhan Sanying Biotechnology Co., Ltd., Wuhan, China); Cell Counting Kit-8 (CCK-8; Dojindo, Kumamoto, Japan); and calcein-AM/propidium iodide (Yeasen Biotechnology, Shanghai, China).

### 2.3. Models of Acute Radiation Intestinal Injury and CBP Administration In Viv*o*

The mice were acclimated for 1 week and then randomly assigned to the following groups (*n* = 6 each): the normal control (NC) group, model group, CBP group and preCBP group. PreCBP refers to administration of the KYNU inhibitor CBP 24 h prior to irradiation, while CBP indicates postirradiation administration. The prevention group (CBP group) and the model group were started on daily intraperitoneal injections of CBP (25 mg/kg/day) or phosphate buffer solution before total abdominal irradiation (TAI), lasting for 3 days [6]. All mice were anesthetised with pentobarbital sodium (50 mg/kg) and then exposed to one TAI dose of 13 Gy of *X*-rays at a rate of 200 cGy/min. The irradiation field ranged from the xiphoid process to the symphysis pubis. At the end of the experiment, mice were anesthetized with pentobarbital sodium for blood collection from the orbital venous plexus. Thereafter, colon tissues were harvested; some of these were fixed in 4% paraformaldehyde and the remainder was stored at −80°C.

### 2.4. Western Blotting

The protein content of the colon tissue was determined using, bicinchoninic acid protein kits (Thermo Fisher Scientific Inc., Waltham, MA, USA). Protein samples were resolved using 10% sodium dodecyl sulfate-polyacrylamide gel electrophoresis and transferred to polyvinylidene fluoride membranes. Nonspecific antigen binding was blocked with 5% skim milk at room temperature (RT: ~25°C) for 2 h, followed by overnight incubation of the membranes at 4°C with the following 1:1000-diluted primary antibodies: anti-β-actin, anti-JNK, anti-phospho-JNK, anti-phospho-p38, anti-p38, anti-phospho-ERK, anti-ERK, and anti-KYNU. The membranes were washed three times for 15 min each on the following day with tris buffered saline containing Tween 20 and incubated with 1:1000-diluted horseradish peroxidase-conjugated secondary antibodies at RT for 2 h. Protein bands were washed and visualized using the ECL Plus chemiluminescence detection system (Thermo Fisher Scientific Inc.) and intensity was analyzed using ImageJ software (v1.8.0, National Institute of Mental Health).

### 2.5. Histological Analysis

Colon tissue samples were fixed in 4% paraformaldehyde, embedded in paraffin, and cut into 5 μm-thick sections that were stained with hematoxylin and eosin (HE) and assessed by optical microscopy. Colon tissues were histopathologically scored using blinded evaluation [7] ranging from 0 to 4: normal colon mucosa (0); minor lesions associated with radiation (1); mild injury characterized by mild inflammation or glandular changes (2); moderate injury characterized by substantial inflammation or loss of colonic epithelial cells (3); severe injury characterized by colonic ulceration or necrosis (4). All scores were averaged to provide semiquantitative histological indicators of colon damage.

### 2.6. Immunohistochemical Analysis

Colon tissue samples were fixed, embedded in paraffin, cut into 5 μm-thick sections, dewaxed, and dehydrated. Antigens were retrieved and nonspecific binding was blocked, using endogenous peroxidase and normal goat serum. The sections were incubated overnight at 4°C with 1:200-diluted anti-KYNU antibody (Abcam) followed by 1:500-diluted biotinylated secondary antibody at RT for 20 min. Finally, images were obtained using an optical microscope (Nikon, Japan).

### 2.7. Terminal Deoxynucleotidyl Transferase dUTP Nick End Labeling (TUNEL) Assay

Apoptosis in colon tissues was detected using a TUNEL assay kit according to the manufacturer's protocol. Briefly, paraffin-embedded sections were dewaxed, rehydrated, and treated with proteinase K for antigen retrieval. After incubation with TUNEL reaction mixture, sections were counterstained with DAPI. Apoptotic nuclei showed green fluorescence under a fluorescence microscope, while all nuclei were stained blue. Apoptotic levels were quantified by counting TUNEL-positive cells in five randomly selected high-power fields (× 400).

### 2.8. RNA Sequencing (RNA-seq)

Total RNA was extracted from colon tissues using TRIzol reagent, and its purity was assessed by spectrophotometry. RNA integrity and quality were further evaluated using the Agilent 2100 Bioanalyzer system. RNA-seq libraries were prepared using the NEBNext Ultra RNA Library Prep Kit for Illumina (New England Biolabs, San Diego, CA, USA) according to the manufacturer's instructions. Library fragments of 300–500 bp were selected and subjected to high-throughput sequencing on an Illumina platform with a read length of 150 bp.

Differentially expressed genes (DEGs) were identified based on the criteria of |log_2_(fold change)| ≥ 1 and *p* ≤ 0.05. DEGs were visualized using, volcano plots and heatmaps. Functional enrichment analyses, including Reactome, Gene Ontology (GO), and Kyoto Encyclopedia of Genes and Genomes (KEGG) pathway analysis, were performed to explore the biological functions and signaling pathways associated with the identified DEGs.

### 2.9. Quantitative Reverse Transcription-Polymerase Chain Reaction (qRT-PCR)

Total RNA was extracted from colon tissues using TRIzol reagent and reverse-transcribed into complementary DNA (cDNA) using a commercial reverse transcription kit (Vazyme Biotech, Nanjing, China), following the manufacturer's protocol. Quantitative real-time PCR was performed using the CFX96 Real-Time PCR Detection System (Bio-Rad Laboratories, Hercules, CA, USA) in combination with SYBR Green qPCR Master Mix (Vazyme Biotech). The primer sequences for the target gene and internal reference gene β-actin are as follows: Human: KYNU, 5 '- GCTCACAACTACTTCACGGA-3′, 5 '- CCCCACTGAACAGGATCG-3′; β-actin: 5′-GGACTTCGAGCAAGAGATGG-3′, 5′-AGCACTGTGTTGGCGTACAG-3′; mice: Kynu: 5′-GTCAAGCCTGCGTTAGTGG-3′, 5′-GGAGGGTTTGAAATTCGGAATCC-3′; β-actin: 5′-GGCTGTATTCCCCTCCATCG-3′, 5′-CCAGTTGGTAACAATGCCATGT-3′. Gene expression levels were normalized to β-actin as the internal control and calculated using the 2^−ΔΔCT^ method.

### 2.10. Fluorescein Isothiocyanate (FITC)-Glucan Permeability Test

Mice, subjected to fasting overnight, were orally administered 400 mg/kg FITC glucan (Sigma-Aldrich Corp). After 4 h, blood samples were collected and serum was separated by centrifugation (4000 rpm, 15 min, 25°C). Fluorescence intensity was measured using a fluorescence spectrophotometer at excitation and emission wavelengths of 485 and 535 nm, respectively.

### 2.11. Cell Culture

The human colon epithelial cell line NCM460 (China General Microbiological Culture Collection Centre; CGMCC) was cultured in Dulbecco's Modified Eagle's Medium containing 10% foetal bovine serum, 100 U/mL penicillin, and 100 U/mL streptomycin at 37°C under a 5% CO_2_ atmosphere. The cells were irradiated with 8 Gy (2 Gy/min) of *X*-rays using a linear accelerator (Varian) to establish a model of RIII in vitro. The irradiated cells were incubated with CBP for 6 h, followed by incubation with propidium iodide, Annexin V, or dihydroethidium fluorescent probes before flow cytometry.

### 2.12. CCK-8 Assay

Cell viability was assessed using CCK-8 assay kits (Dojindo, Japan). Adherent cells were trypsinised, and 5000 cells/well were seeded in 96-well plates and incubated for 24 h to allow adherence. Irradiated cells were incubated with CBP at 37°C under a 5% CO_2_ atmosphere for 6 h. Subsequently, CCK-8 reagent (10 µL) was added to each well, and cells were incubated in darkness for 2 h. Cell viability was determined by measuring absorbance at 450 nm using an enzyme labeler.

### 2.13. Small Interfering RNA (siRNA) Transfection

Synthetic KYNU siRNA was used to suppress *KYNU* expression in intestinal epithelial cells. siRNA was purchased from GenePharma Co., Ltd. (Suzhou, China). The siRNA sequences are as follows: si-NC: 5′-UUC UCC GAA CGU GUC ACG UTT-3′, 5′-ACG UGA CAC GUU CGG AGA ATT-3′; si-KYNU: 5′-CAUUGCGGCUGAACUCAAATT-3′, 5′-UUUGAGUUCAGCCGCAAUGTT-3′; si-KYNU#2:5′-CUCUUGGCCUUCAACCAAATT-3′, 5′-UUUGGUUGAAGGCCAAGAGTT-3′. NCM460 cells were seeded in a 6-well plate at a density of 2 × 10^5^ cells and incubated for 24 h. Once the cell density reached 60%–70%, the siRNA was mixed with Lipofectamine 3000 (Thermo Fisher Scientific, USA) and DMEM (Gibco, USA), and then incubated for 20 min. The mixture was then added to the 6-well plate. The relative expression of KYNU was evaluated after 72 h of incubation, using western blot and quantitative real-time PCR.

### 2.14. Short Time-Series Expression Miner (STEM) Analysis

Gene clustering profiles were obtained using the STEM clustering algorithm to identify temporal gene expression profiles, with the maximum number of model profiles set to 50 and maximum unit change in model profiles between time points set to 3.

### 2.15. Statistical Analysis

All data were statistically analyzed using GraphPad Prism 10 (GraphPad Software Inc., San Diego, CA, USA). Results are presented as mean ± standard deviation (SD). For comparisons involving more than two independent groups (≥ 3), one-way analysis of variance (ANOVA) was performed to assess overall group differences, followed by Tukey's multiple comparisons test to determine pairwise differences between groups. For comparisons between two groups, an unpaired two-tailed Student's *t*-test was used. A *p* − value < 0.05 was considered statistically significant (*⁣*^*∗*^*p*  < 0.05, *⁣*^*∗∗*^*p*  < 0.01, and *⁣*^*∗∗∗*^*p*  < 0.001).

## 3. Results

### 3.1. KYNU Expression Increases in Mice With RIII

**Figure 1:**
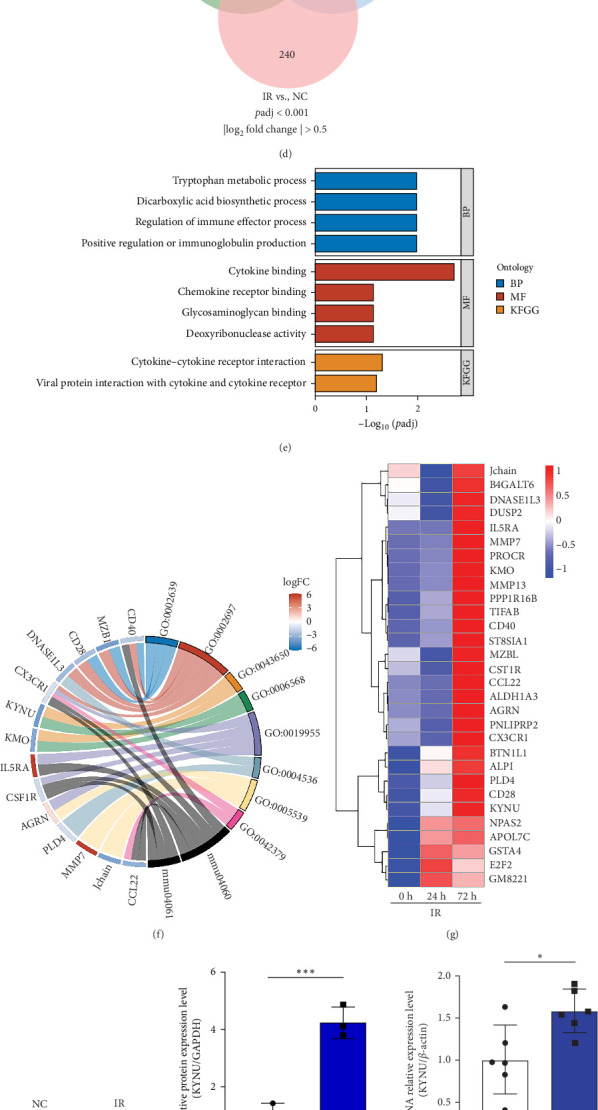
KYNU expression is significantly upregulated in radiation-induced intestinal injury (RIII). (a) Principal component analysis (PCA) of transcriptomic profiles from colon tissues showed clear separation between the normal control (NC) and irradiated (IR) groups. (b) Volcano plot of differentially expressed genes (DEGs) between NC and IR groups, with red and blue dots representing significantly upregulated and downregulated genes, respectively (|log_2_FC| > 1, *p*  < 0.05). (c) Short Time-series Expression Miner (STEM) analysis of two radiation-induced intestinal injury datasets from the GEO database identified temporally upregulated gene expression profiles. (d) Overlap analysis of upregulated genes from the GEO datasets and DEGs from our IR model. (e) GO and KEGG pathway enrichment analyses of the 30 overlapping DEGs. (f) Chord diagram displaying the enriched pathways associated with the overlapping genes. (g) Heatmap showing differential expression of key genes, with KYNU markedly upregulated in the IR group. (h,i) Western blot and quantification showing significantly increased KYNU protein levels in colon tissues of IR mice compared to NC mice (*n* = 3 per group). (j) qPCR analysis confirmed significant upregulation of Kynu mRNA in the IR group. (k) Immunohistochemical staining revealed enhanced KYNU expression in colon sections from IR mice. Data are presented as mean ± SD. *⁣*^*∗*^*p*  < 0.05, *⁣*^*∗∗*^*p*  < 0.01, and *⁣*^*∗∗∗*^*p*  < 0.001.

We performed transcriptome sequencing on colon tissues from the NC group and the radiation model (IR) group to identify key genes involved in the development of RIII. Principal component analysis (PCA) revealed clear separation between the radiation and control groups, indicating significant differences in gene expression profiles at the transcriptomic level (Figure 1a). Volcano plots identified 318 significantly upregulated and 760 downregulated genes in the gut of mice in the IR, compared with those in the NC group (Figure 1b). The STEM tool was used to identify significant temporal expression profiles and associated genes in two RIII mouse data sets in the Gene Expression Omnibus database. Of the identified genes, the upregulated genes were further analyzed (Figure 1c,d). The KEGG analysis and GO analysis and cluster heat maps indicated substantial differences between the KYNU expression in the colons of mice in the NC and IR groups (Figure 1e–g). Western blot and qPCR analyses further confirmed that KYNU protein and mRNA levels were significantly upregulated in the colon tissues in the IR group (Figure 1h–j).

Histochemical staining indicated that KYNU expression was substantially increased in the colons of mice with RIII compared with that in the controls (Figure 1k). Together, these findings suggested that elevated KYNU expression may be associated with RIII pathogenesis.

### 3.2. *KYNU* Knockdown Exerts Protective Effects Against RIII

**Figure 2:**
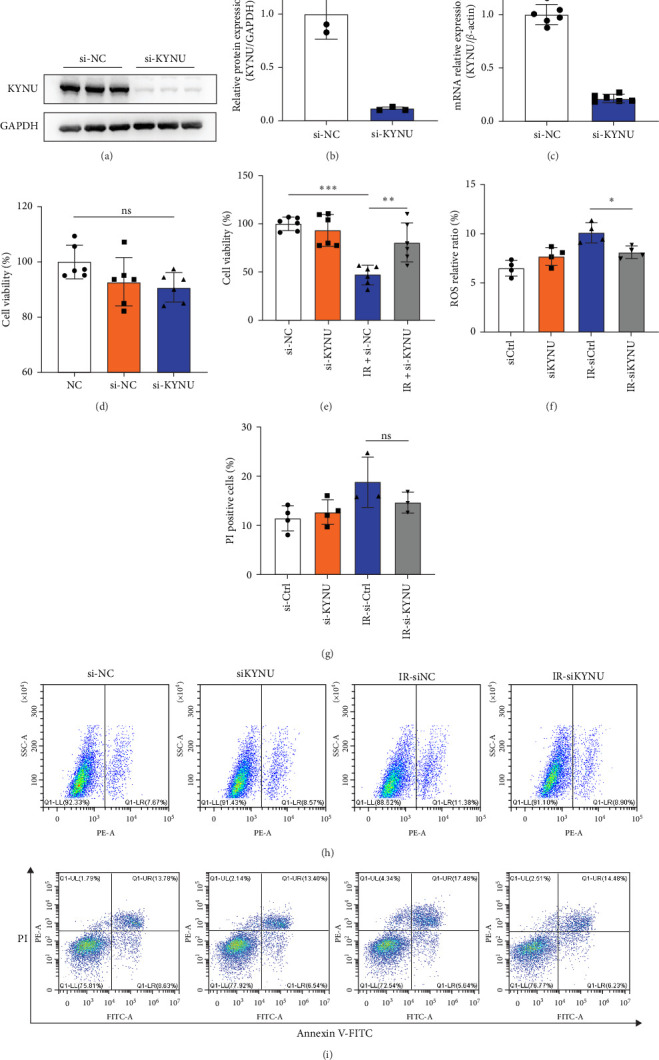
KYNU knockdown alleviates radiation-induced damage in intestinal epithelial cells. (a–c) Western blot (a–b) and qPCR (c) analyses confirmed that siRNA effectively reduced KYNU protein and mRNA expression levels in NCM460 cells. (d) CCK-8 assay showed that KYNU knockdown had no significant effect on cell proliferation under nonirradiated conditions. (e) Under irradiation, KYNU knockdown significantly enhanced the viability of NCM460 cells, suggesting a potential radioprotective effect. (f) Flow cytometry-based quantification indicated that KYNU knockdown markedly reduced intracellular reactive oxygen species (ROS) levels after irradiation. (g) Annexin V/PI double staining and flow cytometry revealed that si-KYNU significantly decreased radiation-induced apoptosis. (h) Flow cytometry plots showed decreased ROS levels in si-KYNU-transfected cells. (i) Annexin V/PI flow cytometry plots further confirmed that KYNU knockdown reduced the proportion of apoptotic cells following irradiation. CCK-8, Cell Counting Kit-8; IR, irradiation; KYNU, kynureninase; ROS, reactive oxygen species. Data are presented as mean ± SD. *⁣*^*∗*^*p*  < 0.05, *⁣*^*∗∗*^*p*  < 0.01, *⁣*^*∗∗∗*^*p*  < 0.001; ns, not significant.

The role of KYNU in RIII was further investigated using the human intestinal epithelial cell line NCM460. We knocked down KYNU using siRNA in NCM460 cells (Figure 2a–c). siRNA has no significant effect on the proliferation of normal cells, but can significantly protect NCM460 cells from RIII (Figure 2d,e). Furthermore, detection of reactive oxygen species (ROS) levels demonstrated that si-KYNU markedly attenuated irradiation-induced oxidative stress in cells (Figure 2f,h). As apoptosis is a major form of irradiation-induced cell death, we performed flow cytometry to assess apoptosis in NCM460 cells 6 h after irradiation. si-KYNU significantly reduced the rate of apoptosis in irradiated cells, confirming its protective effect against radiation-induced apoptosis (Figure 2g,i).

These findings indicate that si-KYNU effectively mitigates radiation-induced cellular injury. To further validate our results, we employed a second independent siRNA targeting KYNU. Consistently, si-KYNU#2 also reduced radiation-induced cellular damage (Supporting Information 1: Figure S1a–i).

In summary, KYNU knockdown promoted cell proliferation and inhibited both apoptosis and oxidative stress following irradiation, indicating its protective role in RIII.

### 3.3. KYNU Is Closely Related to Mitogen-Activated Protein Kinase (MAPK) SignalingSignaling Pathway-Related Protein Expression

**Figure 3:**
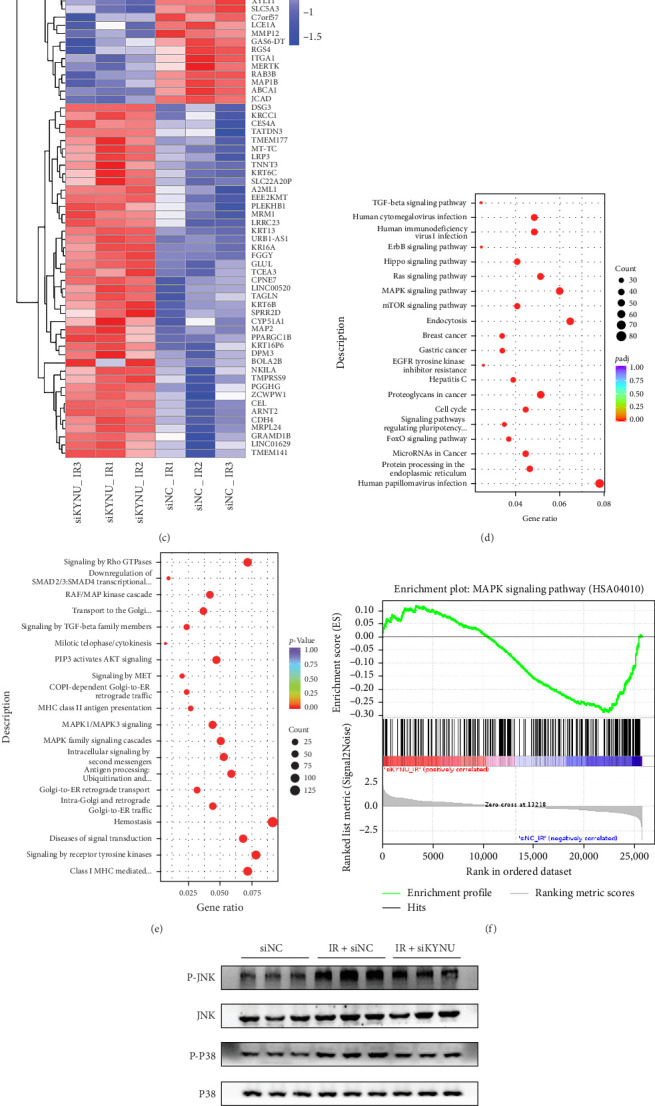
KYNU regulates the expression of MAPK pathway-related proteins in irradiated intestinal epithelial cells. (a) Principal component analysis (PCA) showing distinct gene expression profiles between IR + si-KYNU and IR + si-NC groups. (b) Volcano plot of differentially expressed genes (DEGs). (c) Heatmap displaying clustering of DEGs after KYNU knockdown. (d, e) KEGG and Reactome pathway enrichment analyses highlighting significant enrichment of DEGs in the MAPK signaling pathway. (f) Gene set enrichment analysis (GSEA) demonstrating significant downregulation of MAPK pathway genes in the IR + si-KYNU group. (g, h) Western blot and quantification confirming that KYNU knockdown reduces radiation-induced phosphorylation of key MAPK pathway proteins ERK, p38, and JNK in NCM460 cells, consistent with transcriptomic results. GSEA, gene set enrichment analysis; KEGG, Kyoto Encyclopedia of Genes and Genomes; KYNU, kynureninase; MAPK, mitogen-activated protein kinase; PCA, principal component analysis. Data are presented as mean ± SD. *⁣*^*∗*^*p*  < 0.05, *⁣*^*∗∗*^*p*  < 0.01, and *⁣*^*∗∗∗*^*p*  < 0.001.

Transcriptome sequencing was performed using two experimental cell groups, namely IR + si-NC and IR + si-KYNU, to explore the mechanism of KYNU knockout-mediated protection against RIII damage. Analysis revealed 2067 and 2568 DEGs to be significantly upregulated and downregulated, respectively, in the IR + si-KYNU, compared with those in the IR + si-NC group (Figure 3a,b). Cluster heat maps indicated marked differences in gene expression between the IR + si-NC and IR + si-KYNU groups after KYNU knockdown (Figure 3c).

The KEGG and Reactome pathway enrichment findings indicated that some DEGs were enriched in the MAPK signaling pathway (Figure 3d,e). The original *p*-values for the top 20 KEGG and Reactome enriched pathways corresponding to Figure 3d,e are available in Supporting Dataset 1 (Sheet 1: KEGG; Sheet 2: Reactome). Gene set enrichment analysis (GSEA) showed that genes in the MAPK pathway were significantly downregulated in the IR + si-KYNU group (Figure 3f). Western blot analysis revealed that phosphorylation levels of the key MAPK pathway proteins—ERK, p38, and JNK—were significantly increased in irradiated NCM460 cells (Figure 3g,h). However, KYNU knockdown markedly reduced the phosphorylation of all three proteins, which was consistent with the RNA-seq results (Figure 3g,h). To further confirm the specificity of these findings, we designed and validated a second independent siRNA targeting KYNU (si-KYNU#2). si-KYNU#2 also attenuated the phosphorylation of ERK, p38, and JNK (Supporting Information 1: Figure S2a–d). Collectively, these results indicate that KYNU modulates RIII through the MAPK signaling pathway.

### 3.4. CBP Reduces Radiation-Induced Damage in NCM460 Cells

**Figure 4:**
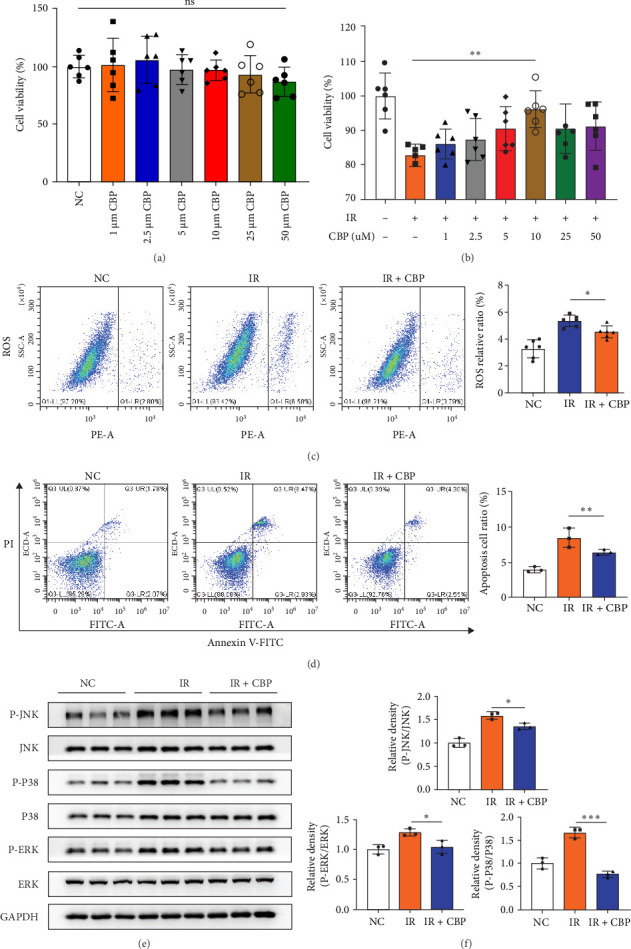
The KYNU inhibitor CBP alleviates radiation-induced damage in NCM460 cells. (a, b) CCK-8 assay showing cell viability of NCM460 cells treated with various concentrations of CBP before (a) and after irradiation (b). (c) Flow cytometry analysis of intracellular reactive oxygen species (ROS) levels demonstrating that CBP significantly suppresses radiation-induced oxidative stress. (d) Annexin V/PI double staining combined with flow cytometry analysis showing that CBP markedly reduces radiation-induced apoptosis. (e, f) Western blot analysis indicating that CBP treatment significantly decreases the phosphorylation levels of key MAPK pathway proteins following irradiation. CBP, carbidopa; ROS, reactive oxygen species. Data are presented as mean ± SD. *⁣*^*∗*^*p*  < 0.05, *⁣*^*∗∗*^*p*  < 0.01, and *⁣*^*∗∗∗*^*p*  < 0.001; ns, not significant.

The KYNU inhibitors CBP and benserazide are commonly used in the treatment of neurodegenerative diseases; however, their potential for treating RIII remains unclear. Given the proinflammatory role of KYNU, we investigated the radioprotective effect of CBP in vitro. We assessed the effect of a range of CBP doses on the viability of NCM460 cells (Figure 4a). CBP also promoted the proliferation of irradiated NCM460 cells, indicating a protective effect against radiation-induced damage in vitro (Figure 4b). The toxicity of CBP ≤ 10 µM was minimal, and its radioprotective effects were most pronounced; hence, 10 μM concentration was selected for subsequent investigation.

Radiation-induced cell death occurs through several steps, among which apoptosis is the most important. We assessed the protective mechanism of CBP using flow cytometry 6 h after irradiation. ROS detection assays indicated that CBP substantially reduced oxidative stress in NCM460 cells (Figure 4c). CBP also substantially reduced apoptosis in the irradiated group, thereby confirming a mitigation effect on radiation-induced apoptosis (Figure 4d). Western blotting showed that CBP also reduced the phosphorylation levels of MAPK pathway related proteins (Figure 4e,f). These results indicate that CBP reduces radiation-induced ROS and apoptosis levels, possibly mainly by lowering the phosphorylation expression of core proteins in the MAPK pathway.

### 3.5. Protective Effect of CBP on RIII in Mice

**Figure 5:**
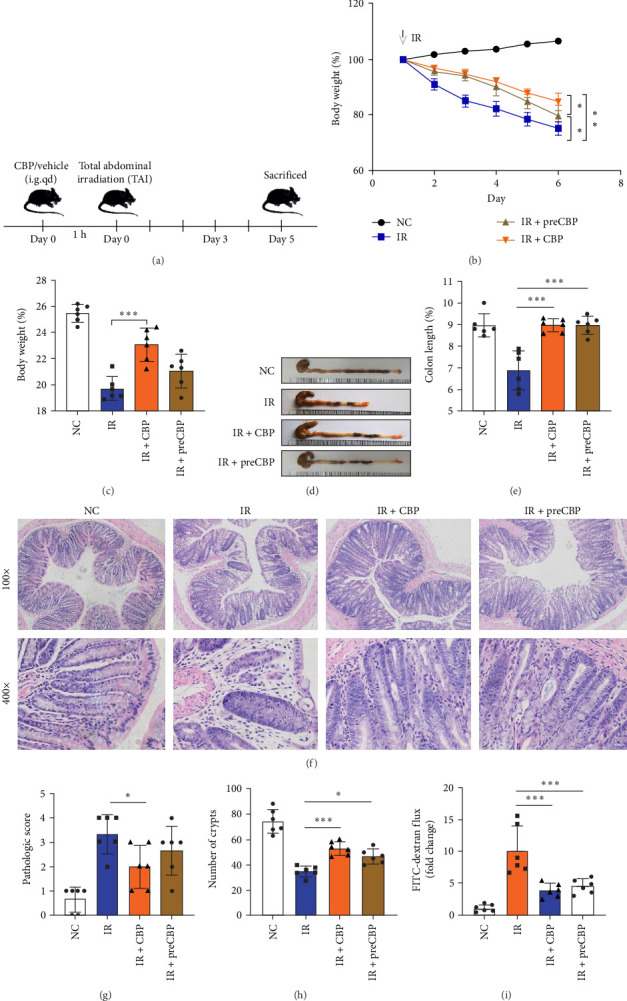
Protective effect of CBP on radiation-induced intestinal injury (RIII) in mice. (a) Schematic illustration of the experimental design evaluating the prophylactic (pre-CBP) and therapeutic (CBP) effects of CBP on mice subjected to total abdominal irradiation (13 Gy). (b, c) Body weight changes during the experiment (b) and final body weight on the last day (c). (d, e) Representative images and quantification of colon length, showing CBP attenuates radiation-induced shortening of the colon. (f) Representative hematoxylin and eosin (HE) staining images of mouse colon tissue illustrating preservation of mucosal architecture and crypt morphology by CBP treatment. (g, h) Histopathological scoring (g) and crypt counts (h) confirming CBP-mediated protection against radiation-induced intestinal damage. (i) Intestinal permeability assessed by FITC-dextran assay demonstrating improved barrier integrity in CBP-treated mice. Data are presented as mean ± SD. *⁣*^*∗*^*p*  < 0.05 and *⁣*^*∗∗∗*^*p*  < 0.001.

To evaluate the therapeutic potential of CBP in RIII, we established a murine RIII model via TAI at a dose of 13 Gy. CBP was administered either prior to (preCBP) or following irradiation (CBP), as illustrated in Figure 5a. Mice in the irradiation group exhibited typical symptoms of RIII, including significant weight loss, reduced activity, and decreased food and water intake (Figure 5b,c). Both prophylactic and therapeutic administration of CBP markedly alleviated these symptoms. The extension of colon length indicated that CBP effectively mitigated the severity of radiation-induced intestinal damage (Figure 5d,e). Histological analysis via HE staining revealed well-preserved mucosal architecture and intact crypts in the control group. In contrast, irradiated mice showed severe crypt loss and disrupted epithelial structure. Notably, CBP treatment restored crypt morphology and increased crypt numbers in the IR + CBP group (Figure 5f). Quantification of the number of crypts and corresponding histopathological scoring further validated the protective effect of CBP (Figure 5g,h). The intestinal barrier integrity was further assessed using FITC-dextran permeability assays, which demonstrated that CBP significantly preserved intestinal barrier function (Figure 5i). Western blot analysis further confirmed that CBP improved the expression of key barrier proteins after radiation, indicating that CBP has a protective effect in maintaining epithelial integrity (Supporting Information 1: Figure S3a–d).

In summary, CBP significantly alleviated RIII in mice by maintaining epithelial integrity, reducing inflammation, and attenuating oxidative stress. These findings suggest that CBP holds promise as a potential therapeutic candidate for the treatment of RIII.

## 4. Discussion

Radiation therapy remains a cornerstone in the treatment of abdominal and pelvic malignancies. However, RIII is a common and debilitating side effect that significantly compromises patients' quality of life and clinical outcomes [7]. Radiation-induced injury to surrounding healthy intestinal tissues can be progressive, potentially leading to serious complications, such as acute gastrointestinal bleeding, perforation, or obstruction. Currently, available treatments for acute RIII are largely palliative in nature, with no effective curative options [8].

KYNU is a key enzyme in the tryptophan–kynurenine metabolic pathway [9]. Aberrant upregulation of KYNU can alter the balance of upstream (kynurenine) and downstream metabolites (e.g., 3-hydroxyanthranilic acid and anthranilic acid), which are closely linked to immune modulation and inflammatory responses [6, 10]. Increasing evidence suggests that KYNU regulates immune homeostasis by orchestrating a switch between immunosuppression and inflammation [11]. Moreover, overactivation of KYNU has been implicated in tumor progression, chemoresistance, and poor prognosis in various cancers [12].

KYNU is primarily expressed in immune cells, where it plays a pivotal role in shaping immune responses [13]. Its expression is significantly upregulated in inflammatory models, such as dextran sulfate sodium-induced colitis. In vitro studies have demonstrated that KYNU silencing suppresses IL-1β-induced proinflammatory cytokine production in intestinal epithelial cells [5]. Similarly, knockdown of KYNU in keratinocytes reduces the expression of psoriasis-associated inflammatory factors in a mouse model of imiquimod-induced dermatitis [6]. Consistent with these observations, our study revealed that KYNU expression was significantly elevated in colonic tissues from irradiated mice. Furthermore, KYNU knockdown enhanced epithelial cell proliferation and attenuated radiation-induced cellular damage and oxidative stress.

Transcriptome analysis, along with KEGG and Reactome pathway enrichment, identified significant alterations in the MAPK signaling cascade upon KYNU knockdown, suggesting KYNU's potential role in mediating RIII via MAPK pathways [14]. MAPKs, including ERK, JNK, and p38, are evolutionarily conserved kinases that regulate cellular metabolism, gene transcription, and immune responses [15, 16]. Western blot analysis showed that radiation-induced phosphorylation levels of proteins, such as ERK significantly increased, and KYNU silencing could reverse this change. These results further support the notion that KYNU promotes RIII progression, at least in part, through MAPK signaling.

KYNU inhibitors, such as CBP and benserazide are FDA-approved drugs commonly used in the treatment of neurodegenerative disorders. However, their efficacy in RIII has not been thoroughly investigated. In our study, CBP administration, whether given prophylactically or therapeutically, substantially alleviated RIII in mice. CBP reduced weight loss, preserved epithelial integrity, and decreased inflammatory cell infiltration. CBP suppressed the secretion of key proinflammatory cytokines, such as TNF-α, IL-1β, and IL-6, all of which contribute to barrier dysfunction and mucosal inflammation [17, 18]. By dampening cytokine release, CBP also likely reduced ROS accumulation, thereby preserving intestinal barrier function and limiting apoptosis [19]. These results suggest that CBP may serve as a promising therapeutic agent for mitigating RIII.

Nonetheless, our study has several limitations. First, the relatively small sample size may have limited the statistical power and generalisability of our findings. Second, there is limited comparative research on the treatment of RIII in existing literature, which makes it difficult for us to systematically evaluate the generalizability of our research results within a broader mechanistic framework. Third, potential confounding factors, such as technical variability or human error, may have influenced certain outcomes. Therefore, further in-depth mechanistic investigations and large-scale validation studies are warranted to confirm the therapeutic potential of CBP in RIII.

## 5. Conclusions

Kynurenine metabolism plays a pivotal role in the pathogenesis of RIII, with KYNU identified as a key regulatory enzyme in this process. Our findings demonstrate that KYNU is significantly upregulated following radiation exposure, contributing to epithelial injury, inflammation, and oxidative stress, partly through activation of the MAPK signaling pathway. Notably, the inhibition of KYNU, either by siRNA or small-molecule inhibitors, such as CBP, effectively mitigated these pathological changes and improved mucosal recovery in vivo. These results provide a theoretical basis for considering KYNU as a promising therapeutic target in RIII. Targeting this pathway may offer a novel strategy to alleviate intestinal damage and enhance the quality of life of patients undergoing abdominal or pelvic radiotherapy.

## Data Availability

Data will be provided upon reasonable request to the corresponding author.

## Nomenclature

KYNU: Kynureninase
MAPK: Mitogen-activated protein kinase
RIII: Radiation-induced intestinal injury
CBP: Carbidopa
CCK-8: Cell Counting Kit-8
TAI: Total abdominal irradiation
HE: Hematoxylin and eosin
DEGs: Differentially expressed genes
FITC: Fluorescein isothiocyanate
RNA-seq: RNA sequencing
KEGG: Kyoto Encyclopedia of Genes and Genomes
RT-PCR: Reverse transcription-polymerase chain reaction
ROS: Reactive oxygen species.

## Supporting Information

Supporting Information 1 Figure S1. Validation of the protective effect of KYNU knockdown using a second independent siRNA. Figure S2. Validation of MAPK pathway protein changes by a second independent siRNA targeting KYNU using western blot analysis. Figure S3. CBP protects against radiation-induced intestinal barrier damage in radiation-induced enteritis.

Supporting Information 2 Supporting Dataset 1. Excel file containing the original p-values for the top 20 pathways from KEGG (Sheet 1) and Reactome (Sheet 2) enrichment analyses, corresponding to Figure 3d, e.
