# Supplementary material for: Targeting Kynureninase Attenuates Radiation-Induced Intestinal Injury via MAPK Signaling Suppression
Source: Mediators Inflamm. 2025 Sep 19;2025:7023259. doi: 10.1155/mi/7023259 (PMC12473747; doi:10.1155/mi/7023259)
Supplement: Supporting Information 1 — Figure S1. Validation of the protective effect of KYNU knockdown using a second independent siRNA. Figure S2. Validation of MAPK pathway protein changes by a second independent siRNA targeting KYNU using western blot analysis. Figure S3. CBP protects against radiation-induced intestinal barrier damage in radiation-induced enteritis. [file 7023259.f1.docx]

Figure S1. Validation of the protective effect of KYNU knockdown using a second independent siRNA. (a) Western blot analysis showing effective reduction of KYNU protein expression by si-KYNU#2; (b) Quantification of KYNU protein levels; (c) Measurement of KYNU mRNA expression following knockdown; (d, e) CCK-8 assay assessing cell viability of si-KYNU#2-treated normal cells (d) and irradiated cells (e);
(f) Statistical analysis of intracellular ROS levels measured by flow cytometry; (g) Quantification of apoptotic cells detected by Annexin V/PI double staining via flow cytometry; (h, i) Flow cytometry plots showing ROS levels (h) and apoptosis rates (i) after si-KYNU#2 treatment. Data are presented as mean ± SD. *p < 0.05, **p < 0.01, ***p < 0.001; ns, not significant.

Figure S2. Validation of MAPK pathway protein changes by a second independent siRNA targeting KYNU using western blot analysis. (a) Western blot analysis showing changes in phosphorylation levels of key MAPK pathway proteins following si-KYNU#2 treatment; (b-d) Quantitative analysis of phosphorylation levels of p-ERK, p-p38, and p-JNK. Data are presented as mean ± SD. *p < 0.05, **p < 0.01.

Figure S3. CBP protects against radiation-induced intestinal barrier damage in radiation-induced enteritis. (a) Western blot analysis showing expression levels of key intestinal barrier proteins after CBP treatment; (b-d) Quantitative analysis of protein expression levels of ZO-1, Occludin, and Claudin-1. Data are presented as mean ± SD. *p < 0.05, **p < 0.01, ***p < 0.001.

**Figure S1**

**
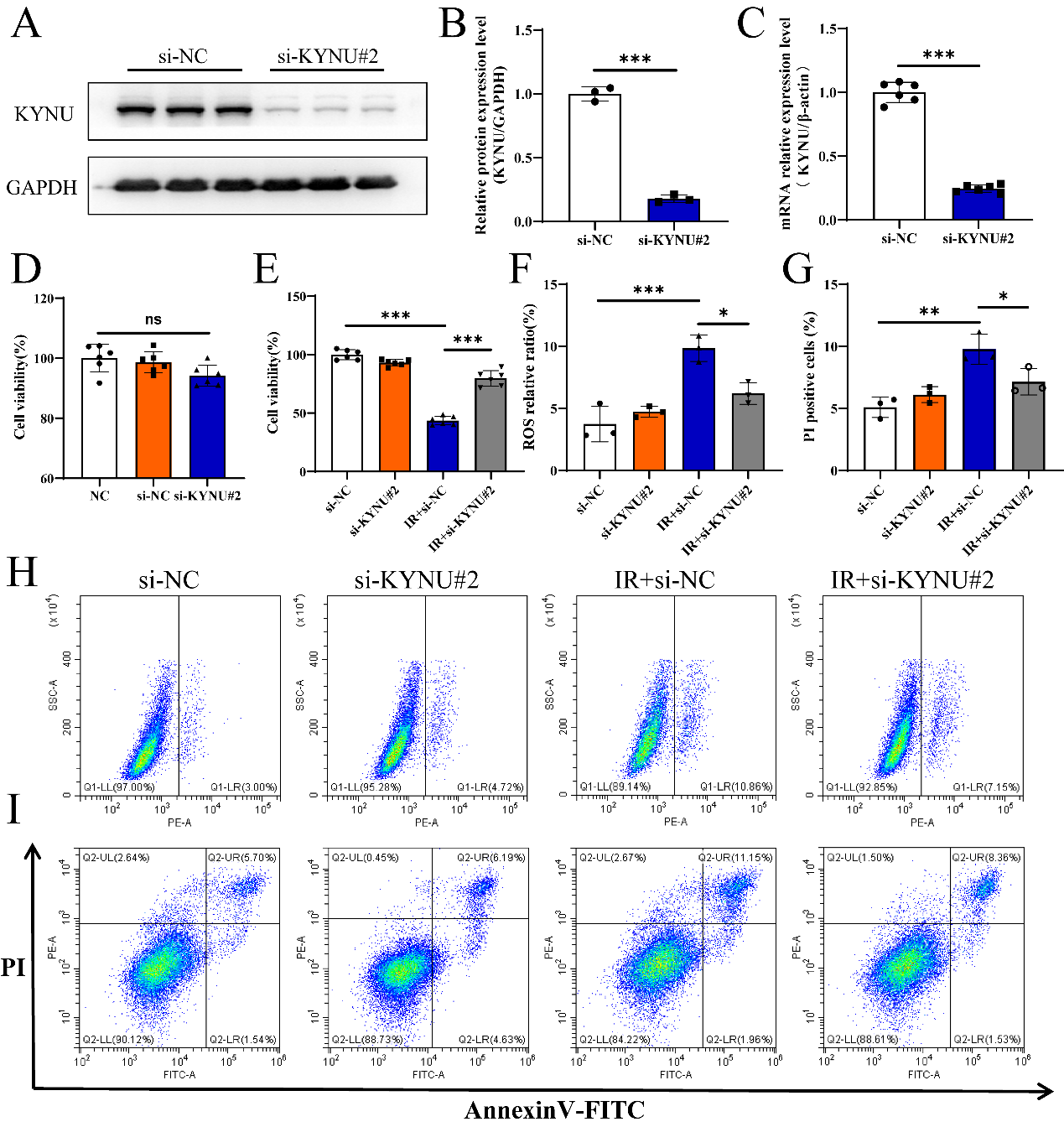
**

**Figure S2**

**
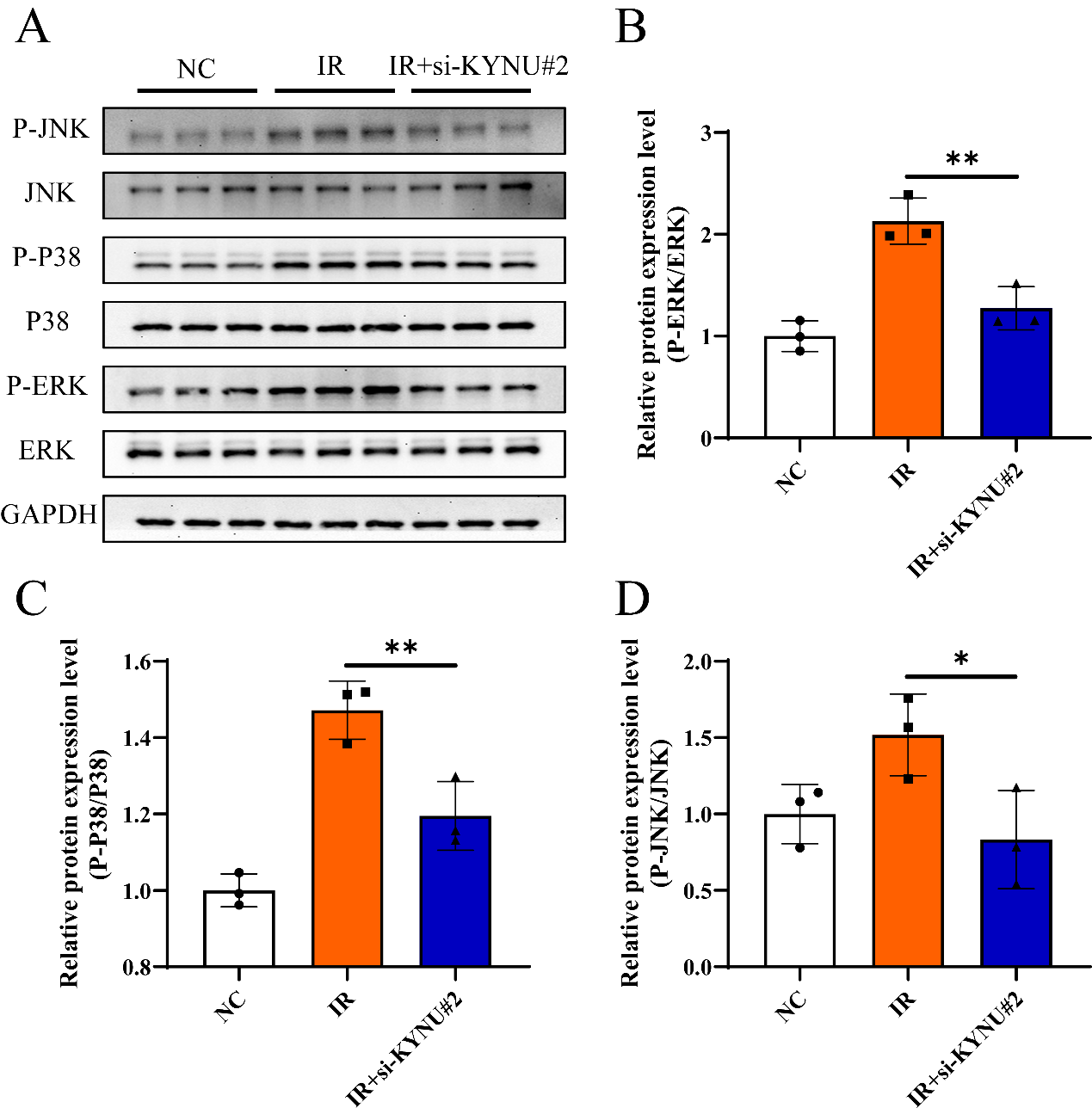
**

**Figure S3**

**
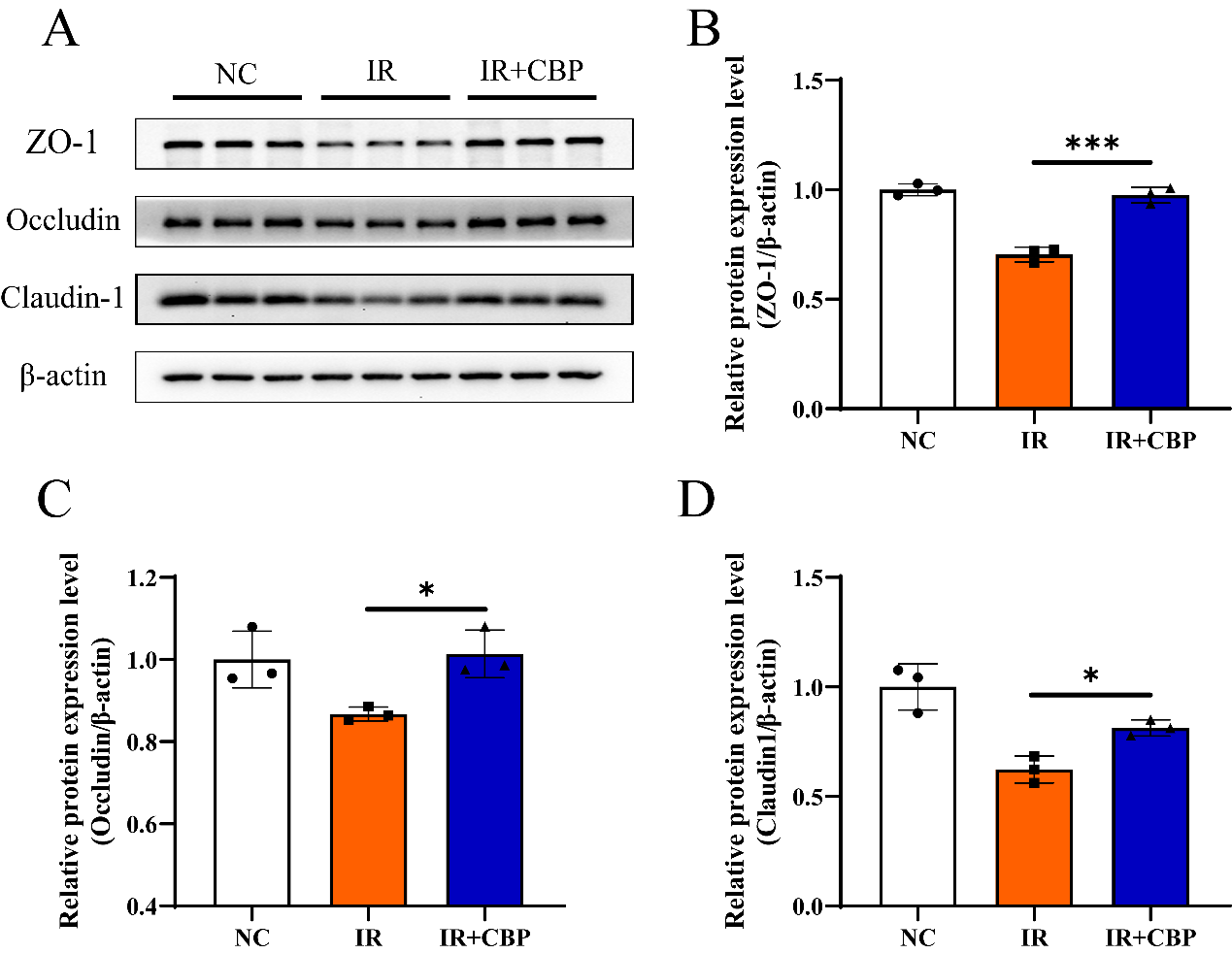
**
